# Ultra low pressure robotic assisted radical prostatectomy (RARP) with pelvic lymph node dissection performed as a day case: first reported case in the UK

**DOI:** 10.1093/jscr/rjag032

**Published:** 2026-01-30

**Authors:** Mayas Rddah, Leo Kretzmer, Gabriel Sullivan, Nicola Milton, Abdoulai Samateh, Edward Calleja

**Affiliations:** Department of Urology, East Sussex Healthcare NHS Trust, Eastbourne District General Hospital, Eastbourne BN21 2UD, United Kingdom; Department of Urology, East Sussex Healthcare NHS Trust, Eastbourne District General Hospital, Eastbourne BN21 2UD, United Kingdom; Department of Urology, East Sussex Healthcare NHS Trust, Eastbourne District General Hospital, Eastbourne BN21 2UD, United Kingdom; Department of Urology, East Sussex Healthcare NHS Trust, Eastbourne District General Hospital, Eastbourne BN21 2UD, United Kingdom; Department of Urology, East Sussex Healthcare NHS Trust, Eastbourne District General Hospital, Eastbourne BN21 2UD, United Kingdom; Department of Urology, East Sussex Healthcare NHS Trust, Eastbourne District General Hospital, Eastbourne BN21 2UD, United Kingdom

**Keywords:** robotic-assisted radical prostatectomy, ultra-low pressure, pneumoperitoneum, same-day discharge

## Abstract

A man in his sixties with locally advanced prostate cancer underwent ultra-low pressure robotic-assisted radical prostatectomy (RARP) with pelvic lymph node dissection (PLND) and was safely discharged the same day. This is the first reported UK case of day-case RARP with PLND performed at 6 mmHg. The patient experienced no complications and reported minimal discomfort postoperatively. We highlight that, with appropriate patient selection, patient’s counselling, perioperative optimization, and ultra-low pressure techniques, same-day discharge after RARP with PLND is feasible, safe, and potentially scalable within NHS pathways.

## Introduction

Radical prostatectomy (RP) remains the cornerstone of management for intermediate risk localized and locally advanced prostate cancer (PC) in patients with life expectancy of >10 years. Since, initially described in 2004, Robotic Assisted Radical Prostatectomy (RARP) has become the gold standard technique for RP, offering reduced hospital admissions and even supporting the feasibility of day-case (DC) RARP [1, 2].

More recently, ultra-low pressure RARP (ulpRARP) has been introduced aiming; as many previous advancements in minimally invasive surgical techniques, to minimizing physiological impact and potentially improving perioperative outcome. In 2023, the first case of RARP with pelvic lymph node dissection (PLND) as a day case was reported in the USA. We aim to report our DC of ulpRARP including PLND. To our knowledge, DC RARP has been reported before, however, this is the first reported case in the UK to be performed under ultralow pressure and include pelvic lymph node dissection as a day case.

## Case report

A gentleman in his 60s with a World Health Organization (WHO) performance status (PS) of 1 was referred via the 2 weeks wait pathway due to elevated prostate specific antigen (PSA) of 7.43 μg/L. His past medical history of chronic obstructive pulmonary disease, hypertension, ankylosing spondylitis and gastroesophageal reflux disease (GORD) for which he is on Amlodipine, Doxazocin, Ramipril, Lansoprazole, and Umeclidinium with Vilanterol inhaler. He does not have any known medical allergies. He is a current smoker of 15 cigarettes per day but does not drink alcohol. His body mass index of 23 kg/m^2^. The American Society of Anaesthesiologists score of 3.

**Figure 1:**
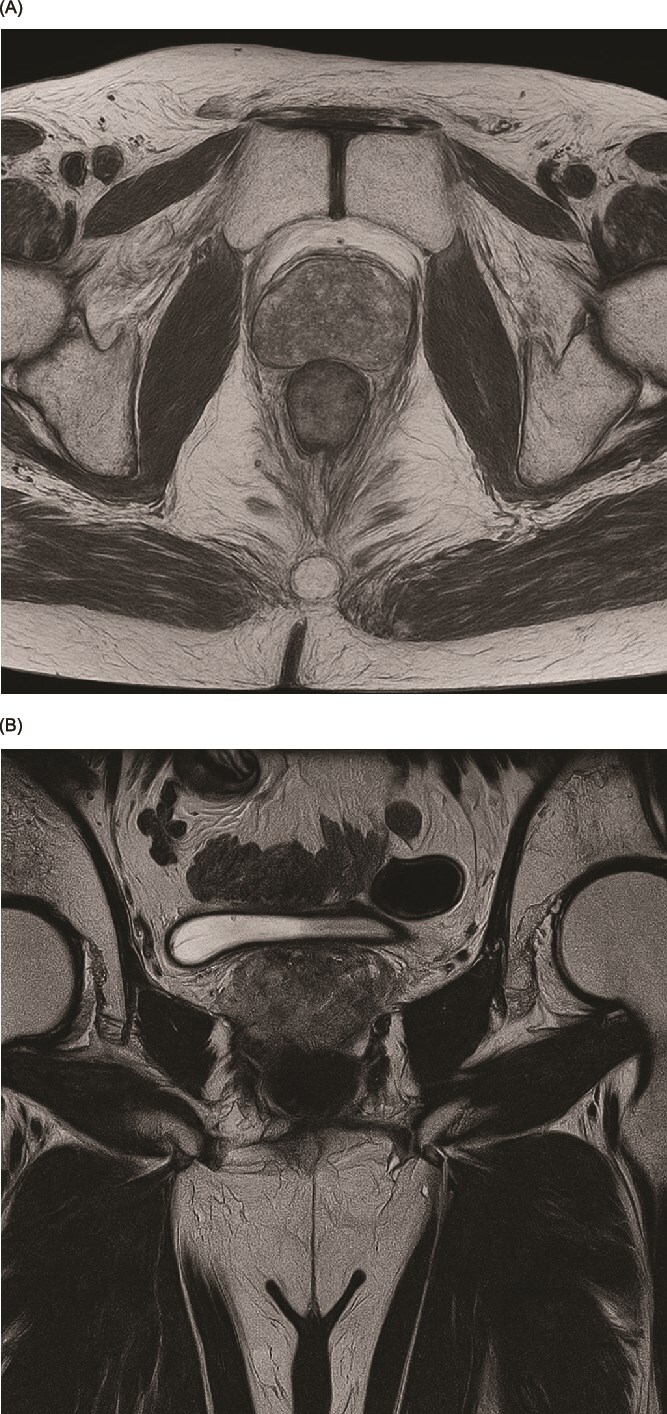
(A, B) Preoperative magnetic resonance imaging (MRI) of the prostate in T2-weighted sequence demonstrating the lesion. (A) Axial view. (B) Coronal view.

Multi-parametric magnetic resonance imaging (MRI) of his prostate (Fig. 1A and B) demonstrated two lesions (PIRAD 4&5). The prostate volume measured 32.83 cc, giving a PSA density of 0.23 ng/ml^2^. No pelvic or para-aortic lymphadenopathy identified and no MRI signal abnormality of the bone marrow. His preliminary MRI staging was T3a N0 M0.

The patient underwent transperineal template biopsies of his prostate. Histopathological analysis demonstrated a Gleason 4 + 5 (Group 5). Cribriform pattern was presented in 8 out of 18 cores with a maximal core involvement of 100% as well as perineural invasion. His computed tomography chest, abdomen and pelvis and bone scan was negative for any metastases.

Following multidisciplinary team discussion, the patient was offered both surgical and radiotherapy options, given the high-grade disease. He opted for RARP over radiotherapy (RT) and androgen deprivation therapy after detailed counselling. He was referred to preoperative preparation included enhanced recovery after surgery (ERAS) and pelvic physiotherapy.

The operation was performed in Trendelenburg position, using the anterior approach, maintaining a working pressure of 6mmhg throughout. The operation commenced with level II PLND (External iliac, internal iliac and obturator). Followed by release of bowel adhesions and mobilization of anterior bladder wall. The anterior bladder neck was incised, both semen vesicles were identified and dissected. The prostate apex was separated. The Cold Extended Maximal Urethral Length Preservation Technique was employed. The anastomosis was completed over 18Fr standard urethral catheter. The console time and the estimated blood loss recorded as 125 min and 150 ml, respectively. The operation was described as of average difficulty with no intraoperative complications. No drains were used.

His recovery was unremarkable. He tolerated oral intake, with no nausea or vomiting, mobilizing well and was discharged later the same day after being educated on self-removal of the catheter which he removed on Day 10 post op. He was given an open access to the emergency urology ward if required.

A telephone follow up the first day post operatively, revealed mild shoulder tip pain and catheter discomfort. He was able to pass flatus and had started deep vein thrombosis prophylaxis as per hospital protocol.

Final histology reported Gleason 4 + 3 with tertiary Gleason 5 prostate adenocarcinoma. Of the twelve lymph nodes sampled, two were positive; his pathological staging was pT3a pN1 with clear margins. His continence improved with pelvic floor exercise, achieved full continence and was discharged from physiotherapy 4 months postoperatively.

His post op PSA was 0.19, 0.11, and 0.22 in May, July, and November, respectively. He was referred to the oncology team for salvage radiotherapy.

## Discussion

The advent of robotic-assisted surgery has significantly transformed the field of urology, offering improved intraoperative and postoperative outcomes, such as reduced pain, decreased blood loss, and shorter hospital stays. Consequently, DC RARP has gained momentum, with accumulating evidence supporting its safety and feasibility in appropriately selected patients [1, 3].

Several institutions, particularly in the United States and France, have reported experience of DC RARP with and without PLND, [1, 4–6]. This is the first published case in the UK to combine ultralow pressure with PLND as day case, a noteworthy milestone within the National Health Service (NHS) surgical pathway.

Historically, PLND has been viewed as a barrier for DC RARP, given its association with prolonged operative time, increased risk of complications (specifically lymphocele development or bleeding), and postoperative monitoring needs [7, 8]. However, our case, consistent with emerging international data [8], demonstrates that PLND does not necessarily preclude a same-day discharge when appropriate patient selection, established perioperative protocols, enhanced recovery, and surgical expertise are applied. Notably, subsequent to this index case, six additional patients at our centre have been planned UlpRARP with PLND as day case, and they were all successfully discharged on the same day, with no readmission. All cases were performed by a single consultant urologist.

Intraoperative strategies were crucial to this success. We utilized ultra-low-pressure pneumoperitoneum of 6 mmHg, a technique increasingly associated with reduced postoperative pain, less shoulder tip discomfort, and quicker recovery [9, 10]. Studies suggested that elevated intra-abdominal pressure can adversely impact cardiopulmonary physiology and immune responses [11, 12], whereas low-pressure insufflation can mitigate these effects facilitating earlier mobilization and discharge. Our experience supports the technical feasibility and safety of RARP at 6 mmHg, reinforcing reports that lower pneumoperitoneum pressures are associated with less postoperative pain and faster recovery [10]. Since 2021, we have performed 352 ultra-low pressure RARP procedures, of which 76 were planned as day cases and 55 were completed successfully; these cases did not include PLND.

A multi-modal approach was integral to achieving these outcomes, incorporating ERAS protocols, minimally invasive techniques, and robust prehabilitation. Key elements included comprehensive preoperative patient counselling, optimized anaesthesia with minimal opioid use, early mobilization, and patient instruction in self-removal of their urinary catheter [6]. Our patient met the selection criteria of low comorbidity burden and high functional status, which have been consistently emphasized in previous feasibility studies [3, 4, 13]. While initial selection was based on morbidity and performance status, our centre now prioritizes robust mental preparation and the availability of a responsible chaperone for 24 hours post-surgery, along with offering 48-hour open access to emergency urological care—a service that has not yet been required.

Available literature demonstrates that DC RARP can be achieved without increased risk of postoperative complications or readmission when strict perioperative protocols are maintained [5, 6]. Our experience supports these findings and suggests that wider adoption of DC RARP within the NHS could reduce hospital stays, alleviate bed pressures, and lower costs, without compromising patient safety.

Importantly, our results align with recent reports indicating that PLND-related complications, such as thromboembolic events, can be minimized through meticulous technique and prompt postoperative mobilization, both of which are achievable within the context of day-case surgery [7]. In this instance, no readmissions, emergency presentations, or complications were observed within a four-month follow-up period, further supporting the safety of this approach.

The median number of lymph node dissection reported is 12–13 per patient which is dependent on pathological report as much as the surgical clearance. The most common sites for metastasis identified is the obturator, internal iliac and external iliac represented by 60%, 58% and 36% respectively [14]. We had 2 subsequent cases where a total of 16 lymph nodes were reported in the final pathology. Having said that, this was performed under ultralow pressure compared to the standard pressure reported before and did not affect the patient being discharged at the same day.

Although these results are encouraging, larger multicentre prospective studies are needed to confirm these findings and to facilitate changes in clinical practice and institutional culture, as well as to further explore cost-effectiveness within different healthcare systems.

## Conclusion

This case adds to the growing body of evidence that day-case ultra-low pressure RARP, including PLND, is both feasible and safe in carefully selected patients. Day-case surgery confers benefits at the individual patient level and on a wider systemic scale, representing a paradigm shift in the management of localized prostate cancer. This approach may serve as a model for continued innovation in minimally invasive urologic oncology within the UK.
